# Engineering of Iron-Based Magnetic Activated Carbon Fabrics for Environmental Remediation

**DOI:** 10.3390/ma8074593

**Published:** 2015-07-22

**Authors:** Hai Haham, Judith Grinblat, Moulay-Tahar Sougrati, Lorenzo Stievano, Shlomo Margel

**Affiliations:** 1 Institute of Nanotechnology and Advanced Materials, Department of Chemistry, Bar-Ilan University, Ramat-Gan 52900, Israel; E-Mails: hai.haham@mail.huji.ac.il (H.H.); judith.grinblat@biu.ac.il (J.G.); 2 Institute Charles Gerhardt (UMR 5253 CNRS), Université Montpellier 2, CC 1502, Place E. Bataillon, Montpellier 34095, Cedex 5, France; E-Mails: Moulay-Tahar.Sougrati@univ-montp2.fr (M.S.); lorenzo.stievano@um2.fr (L.S.)

**Keywords:** thermal decomposition, iron nanoparticles, iron oxide nanoparticles, magnetic nanomaterials, carbon fabrics, environmental remediation

## Abstract

Magnetic Fe_3_O_4_, Fe and Fe/Pd nanoparticles embedded within the pores of activated carbon fabrics (ACF) were prepared by impregnation of the ACF in iron acetylacetanoate (Fe(acac)_3_) ethanol solution, followed by thermal decomposition of the embedded iron precursor at 200, 400 and 600 °C in an inert atmosphere. The effect of the annealing temperature on the chemical composition, shape, crystallinity, surface area, pore volume, and magnetic properties of the various functionalized ACF was elucidated. The Fe nanoparticles within the ACF were also doped with tinier Pd nanoparticles, by impregnation of the Fe/ACF in palladium acetate ethanol solution. The potential use of the functionalized ACF for removal of a model azo-dye, orange II, was demonstrated. This study illustrated the enhanced removal of the dye from an aqueous solution according to the following order: Fe/Pd/ACF > Fe/ACF > ACF. In addition, the enhanced activity of Fe_3_O_4_/ACF in the presence of increasing concentrations of H_2_O_2_ (Fenton catalysts) was also illustrated.

## 1. Introduction

Zerovalent Iron (Fe) and iron oxides (IO) are among the most useful ferromagnetic and ferrimagnetic elements. Fe has the highest magnetic moment at room temperature, a high Curie temperature, and is significantly cheaper than other ferromagnetic elements such as nickel and cobalt [1]. In addition to their unique magnetic properties, IO and Fe nanoparticles are chemically reactive and therefore are good candidates for many environmental remediation applications [2,3].

Previous studies have shown that in the presence of hydrogen peroxide, IO generates free hydroxyl radicals (·OH) that degrade most organic pollutants quickly and non-selectively [4]. Fe nanoparticles were reported to be efficient in the transformation of a wide variety of common organic contaminants such as organic dyes [5], chlorinated organic solvents [6] and fire retardants [7]. Furthermore, Fe nanoparticles doped with an appropriate catalytic metal such as Pd, Pt or Ni demonstrate enhanced elimination activity on many classes of organic pollutants such as azo-dyes and polychlorinated biphenyls [6,8,9].

Preserving the stability of Fe nanoparticles is considered a major challenge, since Fe nanoparticles get oxidized rapidly in water and air, resulting in loss or decrease of magnetism and dispersibility [1,10,11]. To overcome this phenomenon, Fe nanoparticles are usually protected by a protection layer made of polymers [12] carbon [13,14,15], silica [10] or alumina [16]. Previous research showed the efficiency of air-stable iron/carbon (Fe/C) nanomaterials in the removal of heavy metal ions and chlorinated hydrocarbons from water [17,18,19,20]. Such combinations of Fe nanoparticles with the advantages of carbon materials [21], e.g., porous structure and large surface area [22,23], may hold a great potential for adsorptive-reactive water treatment processes [18,24,25,26].

Disposal of dye-contaminated wastewater is a major environmental challenge faced by the textile, dyeing, printing, ink, and related industries [27,28,29]. Dyes are known pollutants that reduce light penetration into water, have an inhibiting effect on photosynthesis, and some are considered toxic and even carcinogenic [30,31].

The present manuscript presents a surfactant-free synthesis which combines the advantages of carbon materials, Fe_3_O_4_ and Fe nanoparticles for environmental applications. Iron oxides and Fe nanoparticles were prepared *in situ* within the pores of activated carbon fabrics (ACF) of a large surface area (1300 m^2^·g^−1^) by thermal decomposition of iron acetylacetanoate, Fe(acac)_3_, embedded in ACF at 200, 400 or 600 °C in an inert atmosphere. The chemical composition, surface area, crystallinity, and magnetic properties of the various functionalized ACF were controlled as a function of the annealing temperature. Doping of the Fe/ACF with smaller Pd nanoparticles was accomplished by reduction of Pd^2+^ ions onto the Fe nanoparticles embedded within the ACF. The removal rate of a model azo-dye, orange II, by the pristine ACF, Fe_3_O_4_/ACF, Fe/ACF, and Fe/Pd/ACF was also studied.

## 2. Experimental Part

### 2.1. Materials

All reagents were purchased from commercial sources and used without further purification, as follows: iron (III) acetylacetanoate, Fe(acac)_3_, (>99.9%), orange II (>85%) and ethanol (Sigma-Aldrich, Rehovot, Israel). Palladium acetate, Pd(acet)_2_, (>95%) (Sterm-Chemicals, Newburyport, MA, USA). Large surface area porous ACF (areal density: 170 g·m^−2^; thickness: 0.6 mm; surface area: 1300 m^2^·g^−1^) (Kynol, Hamburg, Germany).

### 2.2. Synthesis of Iron Oxides and Fe Nanoparticles within the ACF

ACF (240 mg) were cleaned with water and then dried at 120 °C for 30 min. The washed ACF was soaked in 10 mL of 1% Fe(acac)_3_ ethanol solution and then heated at 70 °C for 30 min for the removal of entrapped air and, thereby, enhancing the adsorption of the iron precursor within the ACF pores. After evaporation of the ethanol and cooling to room temperature, the impregnated ACF was soaked in distilled water in order to remove excess reagents, and then annealed at 200, 400 or 600 °C for 4 h in Ar, for the thermal decomposition of the absorbed Fe(acac)_3_. The obtained functionalized ACF were then extensively washed from excess reagents with ethanol and then dried.

### 2.3. Doping Pd Nanoparticles onto the Fe/ACF

Deposition of tinier Pd nanoparticles onto the Fe nanoparticles embedded within the ACF pores was accomplished by reduction of Pd^2+^ onto the Fe surface, as reported previously [9,32]. Briefly, 200 mg of the Fe/ACF were immersed in 10 mL of 0.1% Pd(acet)_2_ ethanol solution. After agitation at room temperature for 10 min, the Fe/ACF were washed from excess reagents with ethanol and then dried.

### 2.4. Characterization of the Various ACF

The thermal behavior of Fe(acac)_3_ was measured by Thermo Gravimetric Analysis (TGA) and Differential Scanning Calorimeter (DSC) (STAR-1 System, Mettler Toledo, Columbus, OH, USA) in an inert atmosphere at a heating rate of 5 °C·min^−1^. Surface morphology was characterized by FEI, Magellan 400 L high-resolution scanning electron microscopy (HRSEM) (JEOL, JSM-840, Tokyo, Japan) of iridium-coated samples. X-ray diffraction (XRD) patterns were recorded using an X-ray diffractometer (model D8 Advance, Bruker AXS, Ness Ziona, Israel) with Cu Ka radiation. ^57^Fe Mössbauer studies were performed using a conventional constant acceleration spectrometer. The velocity calibration was performed using a room temperature α-Fe absorber, and the isomer shift values are given relative to α-Fe. The collected spectra were least-square fitted by appropriate combinations of Lorentzian profiles corresponding to non-equivalent iron environments. Cross-section samples were characterized by high-resolution transmission electron microscope (HRTEM) (JEOL-2100, JEOL, Tokyo, Japan) operating at 200 kV. The HRTEM is integrated with a digital scanning transmission electron microscope (STEM) comprising annular dark and bright field detectors and with a Noran System Six energy-dispersive X-ray spectrometer (EDS) system for elemental analysis. For structural analysis, nanobeam electron diffraction (NBD) and selected area electron diffraction (SAED) techniques were used. Cross-section samples were prepared using focused ion beam (FIB, FESEM; FEI, Helios 600, Hillsboro, OR, USA). For this purpose, a protective layer of platinum was deposited on top of the analyzed material surface. C, H, N and O analysis of the various ACF was performed using an elemental analysis instrument (model FlashEA1112 Instruments, Thermoquast, Austin, TX, USA). Fe and Pd concentrations were determined by inductive coupled plasma (ICP) (ULTIMA-2, HORIBA-Jovin-Yivon, Kyoto, Japan) measurements after dissolving the Fe_3_O_4_, Fe and Fe/Pd compounds embedded within the ACF in concentrated HCl solution. The surface area of the pristine and the various functionalized ACF were measured by N_2_ adsorption-desorption isotherms and calculated according to Brunauer–Emmet–Teller (BET) method [33] (NOVA 3200E Quantachrome, Pale Lane, UK). Total pore volume values of the pristine and functionalized ACF were calculated by measuring the maximum amount of condensed N_2_ adsorbed into the ACF in pressure equilibrium. Knowing the density of the adsorbate, one can calculate the volume it occupies and, consequently, the total pore volume of the various ACF [34]. Room temperature magnetization measurements were performed using Microsense V-VSM vibrating scanning magnometer (VSM) (Microsense, Lowell, MA, USA) with field ranging from −10,000 to 10,000 Oe. Raman spectroscopy (HORIBA Jobin Yvon, Kyoto, Japan) measurements were carried out using an Ar ion laser (power = 10 mW and wavelength = 632.8 nm). The exposure and data acquisition times were 20 and 2 s, respectively. Spectrophotometric measurements were performed using a Cary 100 UV–visible spectrophotometer (Agilent Technologies Inc., Santa Clara, CA, USA).

### 2.5. Environmental Activity

In order to study the use of Fe_3_O_4_/ACF as a Fenton catalyst for decomposition of orange II room temperature kinetics experiments were performed. For this purpose, in each tube a piece of 2 × 1 cm^2^ of Fe_3_O_4_/ACF was agitated in 25 mL of 20 mg·L^−1^ orange II aqueous solution, in the absence and presence of H_2_O_2_ (1%, 2% and 3% w·v^−1^). After each time interval, 0.5 mL of the solution was removed from the solution. The concentration of orange II was then determined by measuring its absorbance at 484 nm. Similar experiments in the absence of H_2_O_2_ were also carried out with the pristine ACF, Fe/ACF and Fe/Pd/ACF. The results of each experiment represent the average of four repetitions with maximal standard deviation of 4%.

## 3. Results and Discussion

### 3.1. Characterization of The Functionalized ACF

**Figure 1:**
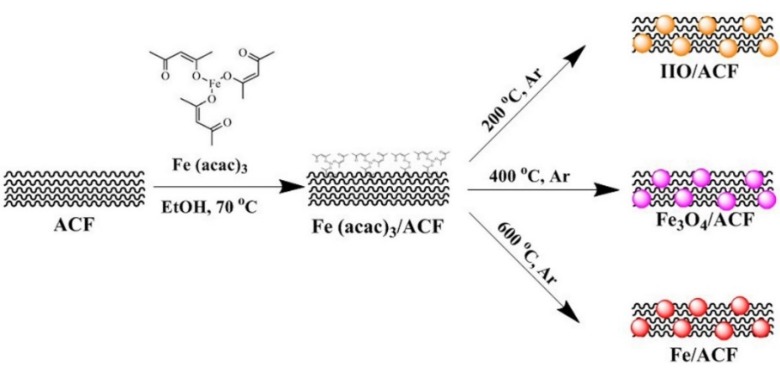
Illustration of the synthetic route of the iron-based functionalized ACF.

Large surface area porous ACF were functionalized with various iron-based nanoparticles for water clean-up applications. A scheme describing the synthetic route of the various nanoparticles is illustrated in Figure 1.

**Figure 2:**
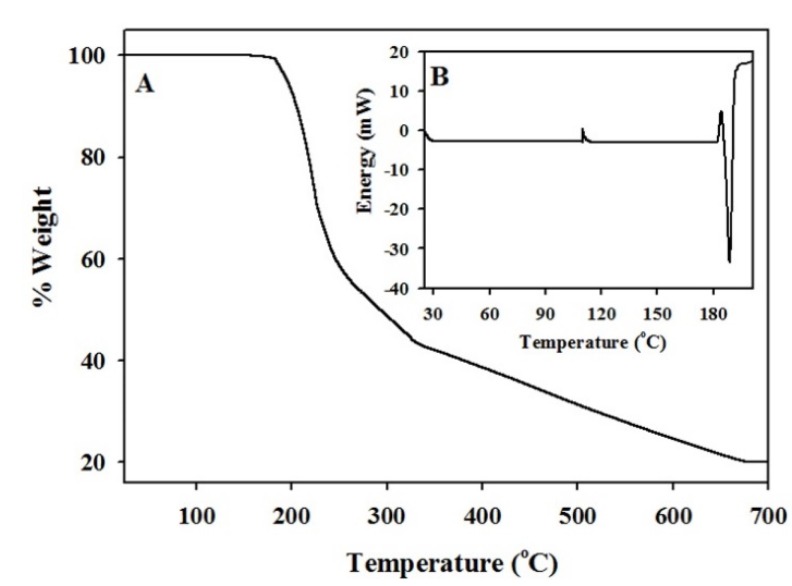
TGA (**A**) and DSC (**B**) thermograms of Fe(acac)_3_ obtained by heating the Fe precursor at a rate of 5 °C·min^−1^ in inert atmosphere.

A TGA thermogram (Figure 2A) of Fe(acac)_3_ indicates that this precursor starts to decompose at 186 °C with 76% weight loss between 186–680 °C. The DSC curve of Fe(acac)_3_ (Figure 2B) is compatible with the TGA behavior displaying an endothermic decomposition peak around 186 °C, as reported in the literature [35]. We also noticed almost a complete thermal decomposition of Fe(acac)_3_ after 4 h of heating at 200 °C in an inert atmosphere.

**Figure 3:**
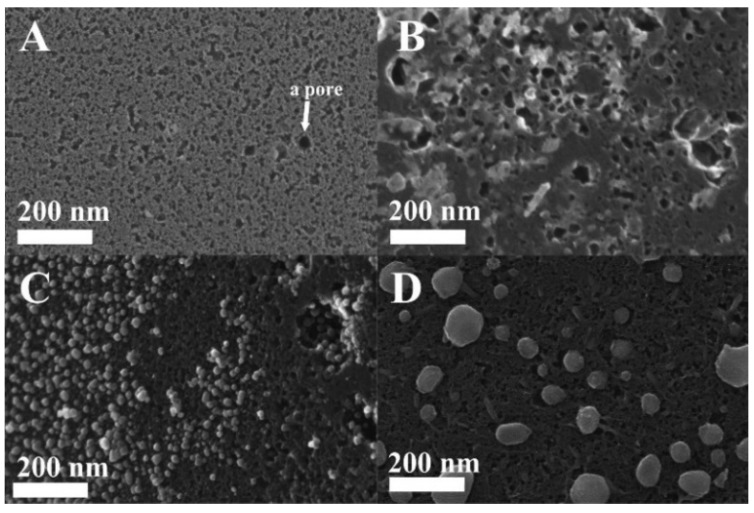
HRSEM images of pristine ACF before impregnation with the Fe(acac)_3_ (**A**); and after impregnation, followed by thermal annealing at 200 °C (**B**); 400 °C (**C**); and 600 °C (**D**) in inert atmosphere according to the experimental section.

Figure 3 exhibits HRSEM images of the non-functionalized (pristine) and the various-functionalized ACF. This figure clearly shows that the annealing temperature of the Fe(acac)_3_/ACF strongly influences the morphology, structure, and distribution of the formed nanoparticles within the various ACF pores. HRSEM image of the pristine ACF (Figure 3A) shows a distinct porous texture. Thermal decomposition of Fe(acac)_3_/ACF at 200 °C (Figure 3B) resulted in a non-homogenous film coating on the ACF. This film coating is probably generated from agglomerated nanoparticles. Thermal decomposition of the Fe(acac)_3_/ACF at 400 °C (Figure 3C) and 600 °C (Figure 3D) exhibit the formation of well-defined nanoparticles embedded within the ACF pores.

**Figure 4:**
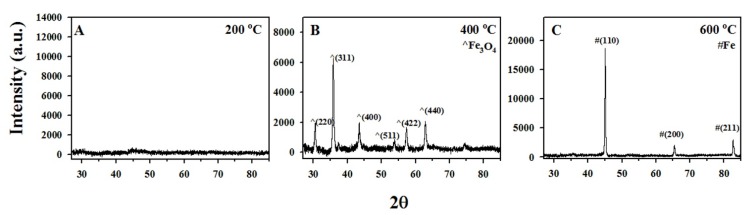
XRD patterns of Fe(acac)_3_/ACF annealed at 200 °C (**A**); 400 °C (**B**); and 600 °C (**C**) for 4 h in Ar prepared as described in the experimental section.

**Figure 5:**
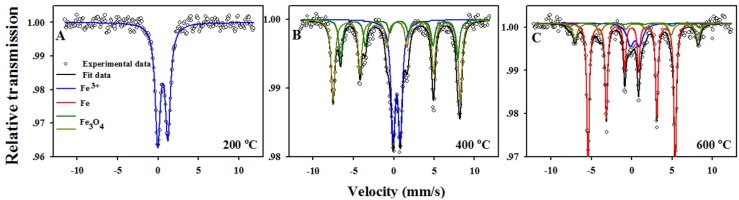
Mössbauer spectra of Fe(acac)_3_/ACF annealed at 200 °C (**A**); 400 °C (**B**); and 600 °C (**C**) for 4 h in Ar prepared as described in the experimental section.

The crystalline and non-crystalline nature of the various-functionalized ACF was investigated by XRD (Figure 4) and Mössbauer spectroscopy (Figure 5). Thermal decomposition of Fe(acac)_3_ within the ACF at 200 °C in Ar resulted in a flat XRD pattern (Figure 4A), indicating the amorphous state of the formed iron phases. Room temperature ^57^Fe Mössbauer spectrum of the same sample (Figure 5A) presents a single quadrupole doublet (blue) which may be attributed to ill-defined paramagnetic IO species [13,36]. The isomer shift and quadrupole splitting of this doublet (0.39 and 0.77 mm·s^−1^, respectively) are characteristic to high-spin Fe^3+^ in oxygen environment [37]. Since many possible nano-sized trivalent iron oxides polymorphs (e.g., α-Fe_2_O_3_, β-Fe_2_O_3_, *etc.*) provide similar hyperfine parameters [38], this iron phase is marked for simplicity as “IIO” (ill-defined iron oxide), as shown in Table 1.

Thermal decomposition of Fe(acac)_3_ within the ACF at 400 °C in Ar resulted in an XRD pattern with diffraction profiles at *2*θ 30.3 (220), 35.4 (311), 43.4 (400), 53.8 (422), 57.5 (511) and 62.5 (440) (Figure 4B). Such pattern corresponds to the spinel structure of Fe_3_O_4_ or γ-Fe_2_O_3_. Mössbauer spectrum of the same sample (Figure 5B) distinguishes Fe_3_O_4_ from γ-Fe_2_O_3,_ showing evidence of partially oxidized Fe_3_O_4_. This Mössbauer spectrum is fitted with three spectral components; two magnetic sextets (orange and green) and one paramagnetic quadrupole doublet (blue). The two sextets have isomer shifts of 0.67 and 0.38 mm·s^−1^, hyperfine fields 450 and 489 kOe and an intensity ratio of about 1:2, respectively. The first sextet is typical for Fe^2.5+^ sites in Fe_3_O_4_ whereas the second sextet is characteristic to Fe^3+^ in both Fe_3_O_4_ and γ-Fe_2_O_3_. The observed intensity ratio indicates a large excess of Fe^3+^ compared to pure Fe_3_O_4_, indicating the presence of a mixture of Fe_3_O_4_ and γ-Fe_2_O_3_. Indeed, the stoichiometry parameter x_m_ = 0.2 (defined as x_m_ = Fe^2+^/Fe^3+^) is characteristic of partially oxidized magnetite with nominal formula Fe_2.8_O_4_. Finally, the paramagnetic doublet is similar to that observed at 200 °C, and can be attributed to the ill-defined IO species mentioned above.Table 1 summarizes the composition of the iron species obtained after annealing of the Fe(acac)_3_/ACF at 400 °C, as follows: 65.2% Fe_3_O_4_ and 34.8% IIO. Recapping the XRD and Mössbauer data suggests that the nanoparticles obtained within the ACF are mainly composed of FCC Fe_3_O_4_ with a unit cell parameter of 8.37 Å.

**Table 1:** Mössbauer spectroscopy parameters and phase composition of the Fe(acac)_3_/ACF annealed at 200 °C (A, B), 400 °C (C, D) and 600 °C (E, F) for 4 h in Ar. IS, QS, LW and H are isomer shift (relative to α-Fe), quadrupole splitting, linewidth, and hyperfine field, respectively.

Temp (°C)	IS (mm·s^−1^)	QS (mm·s^−1^)	LW (mm·s^−1^)	H (kOe)	Composition (wt.%)
200	0.39	0.77	0.47	0	100 IIO
400	0.38	0	0.54	489	65.2 Fe_3_O_4_
	0.67	0	0.54	450	
	0.38	0.88	0.62	0	34.8 IIO
600	0.39	1.15	1.02	0	67.1 Fe
	0.29	0.02	0.65	501	19.2 Fe_3_O_4_
	0.53	0.11	0.65	473	
	0	0	0.35	335	13.8 IIO

Annealing of the Fe(acac)_3_/ACF at 600 °C in Ar resulted in the formation of α-Fe, easily identified by the XRD pattern shown in Figure 4C. In fact, the peaks at *2*θ = 45.1 (110), 65.7 (200) and 83.3 (211) match the crystal plane of cubic BCC structure of α-Fe with a unit cell parameter of 2.84 Å. Mössbauer spectrum of the same sample (Figure 5C) displays the dominant magnetic sextet of α-Fe (red) centered at 0.01 mm·s^−1^. In addition to α-Fe, less-intense magnetically split components at higher fields indicate the presence of Fe_3_O_4_. Also in this case, a minor paramagnetic quadrupole doublet with the hyperfine parameters of IO is present, probably representing ill-defined amorphous IO. The hyperfine parameters of these components are summarized in Table 1. The iron species composition, as presented in Table 1, is 67.1%, 19.2% and 13.8% for α-Fe, Fe_3_O_4_ and IIO, respectively. These findings indicate that the obtained Fe/ACF contains mainly α-Fe phase.

**Figure 6:**
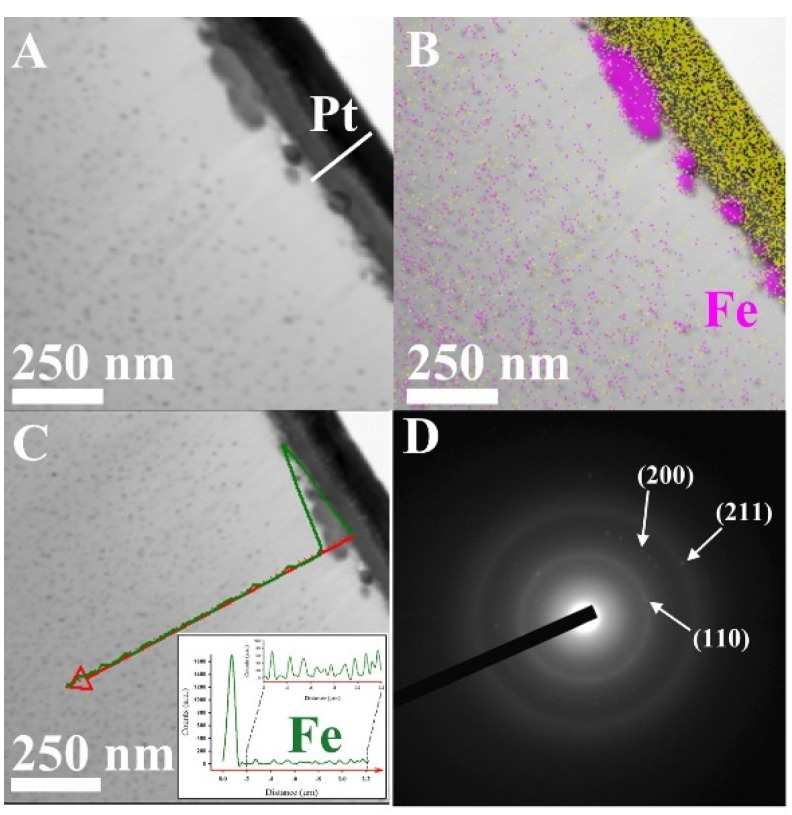
(**A**) A cross-section STEM micrograph of the Fe/ACF coated with a protective layer of Pt, as described in the experimental section; (**B**) elemental mapping of the Fe nanoparticles within the ACF; (**C**) Elemental line-scan profile (red arrow) displaying spatial distribution of the Fe (green) of the cross-section Fe/ACF; (**D**) Selected area electron diffraction of the Fe/ACF.

Further investigation related to the morphology and the structure of the Fe/ACF was obtained by cross-section TEM analysis. For this purpose, the Fe/ACF sample was coated with a protective layer of Pt and then sliced by FIB. Figure 6A,B show that the Fe/ACF cross-section comprising two populations of nanoparticles that vary in size and shape according to their location; at the surface of the ACF and within the ACF core. The line-scan profile of the elemental Fe from the surface of the ACF into its core (Figure 6C) is consistent with the observations by STEM (Figure 6A) and the elemental mapping (Figure 6B), indicating the presence of two populations of nanoparticles, as mentioned above. Additional selected area electron diffraction (SAED) of the Fe/ACF (Figure 6D) shows reflections matching the interplanar spacing of d*_110_*, d*_220_* and d*_211_* of α-Fe (α-Fe, *a* = 2.86 Å, BCC, PDF# No. 04-007-9753).

**Figure 7:**
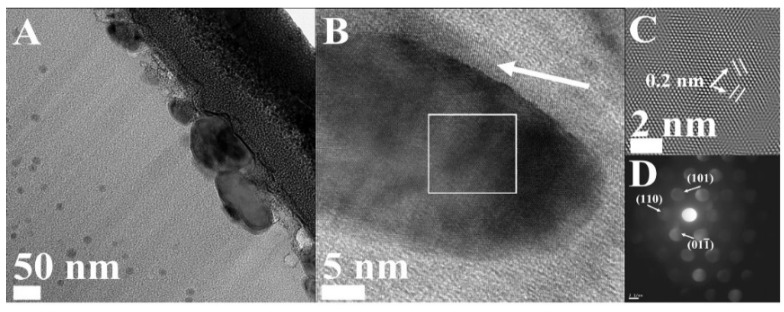
Cross-section TEM and HRTEM analysis of the Fe/ACF samples: (**A**) TEM micrograph of the Fe/ACF surface; (**B**) HRTEM image of a thin IO layer (white arrow) coating the α-Fe nanoparticle; (**C**) Magnified HRTEM image of the area marked by the white square (inset B) displaying lattice fringes of α-Fe; (**D**) NBD taken from 4 nm diameter displaying sets of reflections matching the structure of α-Fe.

TEM micrograph of the nanoparticles at the surface of the ACF (Figure 7A) exhibits wide distributed nanoparticles with average diameter 85 ± 20 nm, depending on the pore size. The data obtained from the HRTEM suggests for these nanoparticles a structure of α-Fe nanoparticle coated with a thin IO layer, as shown in Figure 7B. Figure 7C shows magnified lattice fringes obtained from the area marked by the white square in Figure 7B which match the interplanar spacing *d*_110_ of BCC α-Fe (d = 0.203 nm). In addition, a nanobeam diffraction (NBD) pattern shows the sets of reflections which can be uniquely indexed on the basis of the BCC structure of α-Fe (Figure 7D). Recapping the observed HRTEM data indicate Fe nanoparticles coated with a thin layer of IO at the surface of the ACF.

**Figure 8:**
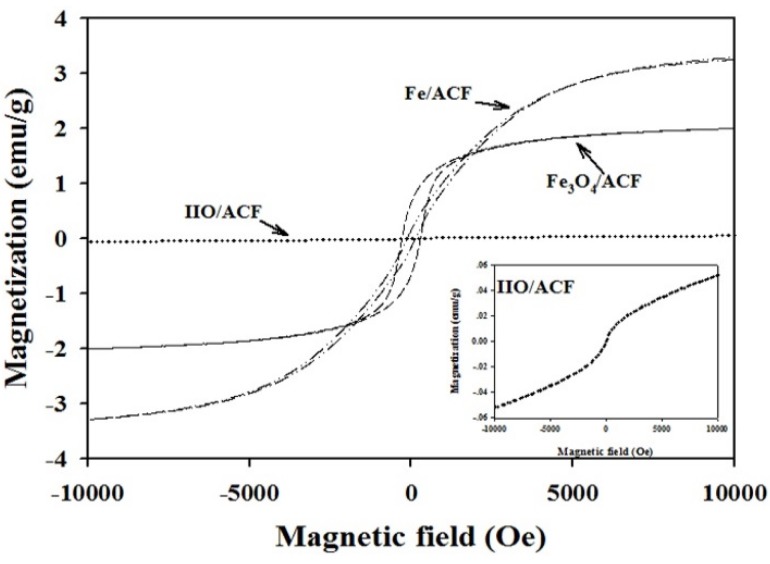
Room temperature magnetization loops of IIO/ACF, Fe_3_O_4_/ACF and Fe/ACF obtained as described in the experimental part. A magnified magnetization loop of the IIO/ACF is also presented in the right inset.

The magnetic properties of the functionalized ACF were studied by room temperature VSM magnetization loops, as shown in Figure 8 and summarized in Table 2. The magnetization loops of Fe_3_O_4_/ACF and Fe/ACF display ferromagnetic response with magnetization saturations (*M_s_*) of 2.00 and 3.25 emu·g^−1^ and coercivity values of 270 and 80 Oe, respectively. It should be noted that the relatively low *M*_s_ values of the Fe_3_O_4_/ACF and Fe/ACF compared to the reported bulk values of the Fe_3_O_4_ and Fe [1], arise from the non-magnetic carbon content, as reported previously [39]. The IIO/ACF magnetization loop displays a magnetic response which is proportional to the applied magnetic field, thus shows no *M_s_*. Supporting this observation, the Mössbauer analysis suggests a paramagnetic phase within the IIO/ACF (Figure 5).

The pristine ACF, IIO/ACF, Fe_3_O_4_/ACF, and Fe/ACF differ in the Fe content, surface area, and pore volume as shown in Table 2. The Fe content of the various functionalized ACF is 11.9%, 10.7% and 9.7% for IIO/ACF, Fe_3_O_4_/ACF and Fe/ACF, respectively. Surface area values of the various ACF decreased from 1292 for the pristine ACF to 572, 718 and 880 m^2^·g^−1^ for the IIO/ACF, Fe_3_O_4_/ACF and Fe/ACF, respectively. The same trend was also observed for the total pore volume values that decreased from 0.70 for the pristine ACF to 0.32, 0.40 and 0.52 cm^3^·g^−1^ for the IIO/ACF, Fe_3_O_4_/ACF and Fe/ACF, respectively. These results arise two main observations: First, the obtained nanoparticles fill the ACF pores and thus decrease the surface area and pore volume of the pristine ACF. Second, the surface area and pore volume increase with the annealing temperature (e.g., 0.32, 0.40 and 0.52 cm^3^·g^−1^ for 200, 400 and 600 °C, respectively). This observation is consistent with the increase in nanoparticle density with increasing temperature, as illustrated in the HRSEM images shown in Figure 3.

**Table 2:** A comparison between the chemical/physical properties of the pristine ACF, IIO/ACF, Fe_3_O_4_/ACF, and Fe/ACF obtained according to the experimental section.

Sample	Fe (wt.%)	Composition (%)	*M_s_* (emu·g^−1^)	Coercivity (Oe)	Surface Area (m^2^·g^−1^)	Pore Volume (cm^3^·g^−1^)	Raman (I_d_/I_g_)
ACF	–	–	–	–	1292	0.70	1.23
IIO/ACF	11.9	100 IIO	–	–	572	0.32	1.29
Fe_3_O_4_/ACF	10.7	65.2 Fe_3_O_4_ 34.8 IIO	2.00	275	718	0.40	1.31
Fe/ACF	9.7	67.1 Fe 13.8 IIO 19.2 Fe_3_O_4_	3.25	80	880	0.52	1.36

**Figure 9:**
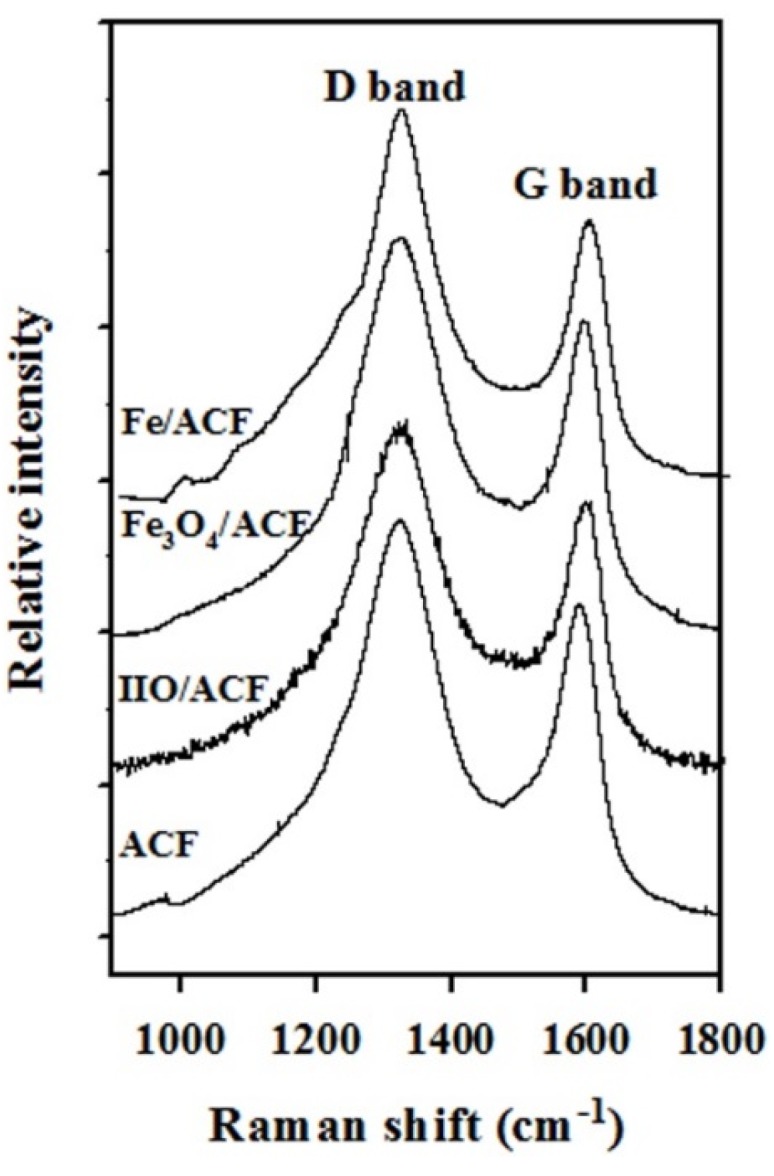
Raman spectra the pristine ACF, IIO/ACF, Fe_3_O_4_/ACF and Fe/ACF obtained as described in the experimental section.

The Raman spectra of pristine ACF and of the various functionalized ACF are presented in Figure 9. All of the ACF possess two main peaks around 1330 cm^−^^1^ (D band) and 1580 cm^−1^ (G band). The G band is associated with the *E*_2g_ stretching vibrations in the basal-plane of graphite [40,41], while the D band is explained as a disorder-induced lattice distortion, or an amorphous C background signal [42]. A comparison between the intensity ratios of the D band (I_d_) and the G band (I_g_) shows an increase of I_d_/I_g_ from 1.23 for the pristine ACF to 1.29, 1.31, 1.36 for the IIO/ACF, Fe_3_O_4_/ACF and Fe/ACF, respectively (Table 2). This probably indicates that increasing the annealing temperature leads to a slight increase in the content of amorphous carbon compared to graphitic carbon.

### 3.2. Doping of The Fe/ACF with Tinier Pd Nanoparticles

**Figure 10:**
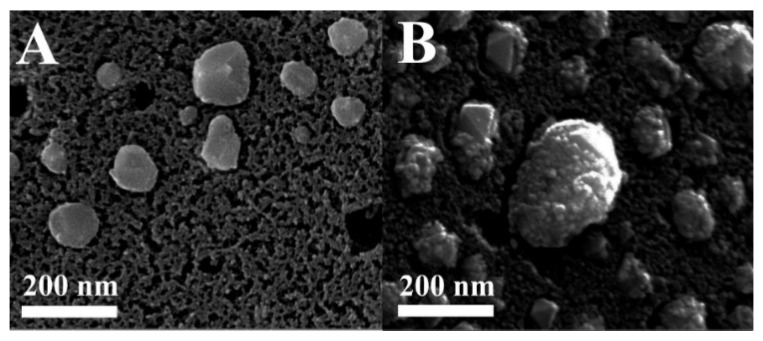
HRSEM images of the Fe/ACF (**A**); and the Fe/Pd/ACF (**B**) prepared as described in the experimental section.

Attachment of smaller Pd nanoparticles onto the surface of the Fe nanoparticles was conducted in order to enhance the environmental activity of the Fe/ACF towards organic pollutants. The deposition of the tinier Pd nanoparticles onto the Fe nanoparticles embedded within the ACF pores was verified qualitatively and quantitatively by HRSEM and ICP measurements. Figure 10 shows HRSEM images of the Fe/ACF (A) and Fe/Pd/ACF (B), illustrating the smaller Pd nanoparticles coating onto the Fe nanoparticles which are embedded within the carbon pores. The measured diameter of the Pd nanoparticles varied from 5 to 14 nm as shown in Figure 9C. The Pd content, as measured by ICP, is 0.9 wt.%.

### 3.3. Environmental Activity-Proof of Concept

#### 3.3.1. Activity of The Fe_3_O_4_/ACF in Absence or Presence of H_2_O_2_

**Figure 11:**
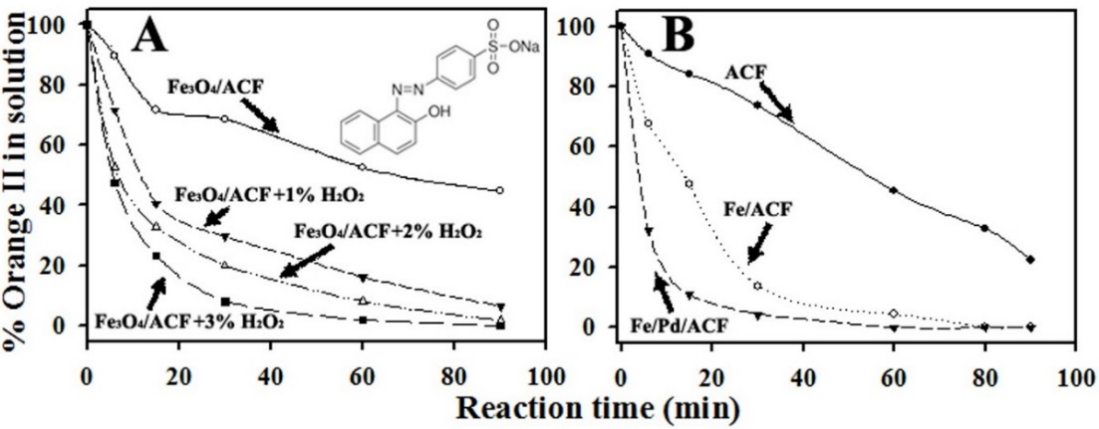
(**A**) Chemical structure and elimination rate at room temperature of orange II by Fenton reaction; (**B**) elimination rate at room temperature of orange II by the Fe/ACF and Fe/Pd/ACF.

The activity of Fe_3_O_4_/ACF as a Fenton catalyst was studied in room temperature on orange II as a model environmental contaminant (Figure 11A). The removal rate of orange II from an aqueous solution by the Fe_3_O_4_/ACF containing 1%, 2% and 3% w/v H_2_O_2_, is significantly higher than that of the pristine ACF and Fe_3_O_4_/ACF in absence of H_2_O_2_. For example, 60 min after initiating the reaction, 84%, 92% and 98% of the initial amount of orange II was reduced by the Fe_3_O_4_/ACF+1%, 2% and 3% H_2_O_2_, compared to 48% by the Fe_3_O_4_/ACF and 52% by the pristine ACF (Figure 11B). As expected, the degradation rate of orange II increases with H_2_O_2_ concentration. This is because the H_2_O_2_ concentration is directly related to the content of ·OH radicals generated in the Fenton reaction. We assume that in this study, as reported previously [43], orange II is totally mineralized by the Fenton reaction.

#### 3.3.2. Activity of Fe/ACF and Fe/Pd/ACF

The activity of Fe/ACF and Fe/Pd/ACF was also studied in room temperature on orange II as a model environmental contaminant (Figure 11B). Both Fe/ACF and Fe/Pd/ACF showed enhanced elimination activity compared to the pristine ACF. As shown in Figure 10B, only 5 min after initiation of the reaction, 68% of the initial amount of orange II was reduced by the Fe/Pd/ACF, compared to 30% and 10% by the Fe/ACF and the pristine ACF, respectively.

We assume that the Fe/Pd/ACF combines the advantageous properties of Fe, Pd and carbon matrix. Previous research have shown that Fe nanoparticles induce the cleavage of azo-bond compounds into amine products that are more amenable to mineralization in biological treatment processes [44]. Moreover, the attachment of an appropriate catalytic metal to Fe, such as Pd in this case, increases degradation rates on azo-compouds [45]. Finally, the adsorptive properties of the carbon matrix, as reported previously [3,24], enable the Fe/Pd/ACF to work in adsorptive-reactive manner. Future research will seek to fully clarify the adsorptive and reactive mechanism and potential use of the Fe/Pd/ACF.

## 4. Conclusions

Fe_3_O_4_/ACF, Fe/ACF and Fe/Pd/ACF were engineered by thermal decomposition of iron acetylacetonate supported on ACF at different temperatures in an inert atmosphere. The physical and chemical properties of the various functionalized ACF were found to be significantly dependent on the annealing temperature of the Fe(acac)_3_/ACF. The potential use of these magnetic fabrics for environmental use was demonstrated with a model azo-dye, orange II. This study illustrated the potential environmental utility of Fe_3_O_4_/ACF in the presence of increasing concentrations of H_2_O_2_. In addition, the catalytic activity of the Fe/Pd/ACF compared to Fe/ACF was also demonstrated. In future research we will examine the catalytic removal efficiency of the various iron-based ACF on a diverse set of contaminants. Of special interest is the Fe/Pd/ACF. The catalytic performances, as well as the mechanistic activity, will be thoroughly studied.
